# Silencing of the IKKε gene by siRNA inhibits invasiveness and growth of breast cancer cells

**DOI:** 10.1186/bcr2644

**Published:** 2010-09-23

**Authors:** Bin Qin, Kun Cheng

**Affiliations:** 1 Division of Pharmaceutical Sciences, School of Pharmacy, University of Missouri-Kansas City, 2464 Charlotte Street, Kansas City, MO 64108, USA

## Abstract

**Introduction:**

IκB kinase ε (IKKε) is a member of the IKK family that plays an important role in the activation of NF-κB. Overexpressed in more than 30% of breast cancers, IKKε has been recently identified as a potential breast cancer oncogene. The purpose of the present study is to examine the therapeutic potential of IKKε siRNA on human breast cancer cells.

**Methods:**

Eight siRNAs targeting different regions of the IKKε mRNA were designed, and the silencing effect was screened by quantitative real-time RT-PCR. The biological effects of synthetic siRNAs on human breast cancer cells were investigated by examining the cell proliferation, migration, invasion, focus formation, anchorage-independent growth (via soft agar assay), cell cycle arrest, apoptosis (via annexing binding), NF-κB basal level, and NF-κB-related gene expressions upon the IKKε silencing.

**Results:**

Silencing of IKKε in human breast cancer cells resulted in a decrease of focus formation potential and clonogenicity as well as *in vitro *cell migration/invasion capabilities. Moreover, knockdown of IKKε suppressed cell proliferation. Cell cycle assay showed that the anti-proliferation effect of IKKε siRNA was mediated by arresting cells in the G_0_/G_1 _phase, which was caused by downregulation of cyclin D_1_. Furthermore, we demonstrated that silencing of IKKε inhibited the NF-κB basal activity as well as the Bcl-2 expression. Significant apoptosis was not observed in breast cancer cells upon the silencing of IKKε. The present study provided the first evidence that silencing IKKε using synthetic siRNA can inhibit the invasiveness properties and proliferation of breast cancer cells.

**Conclusions:**

Our results suggested that silencing IKKε using synthetic siRNA may offer a novel therapeutic strategy for breast cancer.

## Introduction

IκB kinase ε (IKKε, also named IKKi/IKBKE) is a member of the IKK family, which contains five distinct but closely related members: IKKα, IKKβ, IKKγ, TBK-1 and IKKε [1,2]. IKK is an important mediator of the activation of NF-κB, which is a heterodimeric transcription factor playing essential roles in inflammation and cancer pathogenesis. The NF-κB family is composed of Rel A, Rel B, c-Rel, p50/p105 and p52/p100. Inhibitors of kappa B (IκBs) bind to the homodimers or heterodimers of NF-κB proteins and cause their cytoplasmic retention in an inactivated form. Upon stimulation, IκBs are phosphorylated by IKK complexes - which leads to the ubiquitination and proteasomal degradation of IκBs. NF-κB is then released and translocated into the nucleus to regulate the expression of target genes involved in immune and inflammatory responses [3,4]. Discovered in 2000, IKKε shows a 33% and 31% sequence identity with IKKα and IKKβ, respectively, in the N-terminal kinase domain, but has distinct function in the activation of NF-κB pathway [2,5]. Overexpression of IKKε is strongly correlated with the nuclear localization of c-Rel in breast cancer specimens, indicating that a substantial fraction of NF-κB activation is induced by aberrant IKKε in breast cancer cells [6]. The relationship between IKKε and NF-κB, however, is not fully understood [4,7].

IKKε is primarily involved in signaling of inflammatory and immune processes [8,9]. Peant and colleagues reported that overexpression of IKKε in hormone-sensitive LNCaP and 22Rv1 prostate tumor cells induced secretion of numerous inflammatory cytokines, such as IL-8 and IL-6. However, the IKKε-dependent IL-8 and IL-6 overexpressions are not mediated by the activation of NF-κB pathway. Instead, the authors speculated that high IKKε expression leads to nuclear translocation of itself to activate these inflammatory cytokine genes [10]. Recently, the role of IKKε in cancer has been studied by several groups. Sonenshein and colleagues observed for the first time a higher level of IKKε in breast cancer cell lines and specimens, whereas little IKKε expression was detected in normal breast epithelial cells [11]. Furthermore, Boehm and colleagues indentified IKKε as a new potential oncogene in breast cancer cell lines and patient-derived tumors using three complementary genetic approaches. Overexpression of IKKε was observed in over 30% of breast cancer cell lines and carcinomas [4,6,7]. On the other hand, inhibition of IKKε in breast cancer cells with overexpressed IKKε induced cell death [6]. All these up-to-date data strongly support the role of IKKε in tumorigenesis, and subsequently blocking the IKKε expression would be a rational strategy to treat breast cancer.

Among various strategies to inhibit the oncogene expression, RNA interference (RNAi) offers considerable promise for cancer therapy due to its ability to potently knockdown a specific gene. siRNA of 21 to 23 nucleotides in length silences a target gene by binding to its complementary mRNA and triggering its degradation [12,13]. In the present study, we intend to evaluate the effect of silencing IKKε on colonigenicity, invasive properties, proliferation, and apoptosis in breast cancer cells using synthetic siRNA.

## Materials and methods

### Reagents

Lipofectamine-2000 and TRIzol reagent were purchased from Invitrogen Corp. (Carlsbad, CA, USA), Cell culture products were obtained from Atlanta Biologicals, Inc. (Lawrenceville, GA, USA) and Mediatech, Inc. (Manassas, VA, USA). BSA was purchased from Sigma-Aldrich Corporation (St Louis, MO, USA). SYBR Green-1 dye universal master mix and Multiscript RT were purchased from Applied Biosystems, Inc. (Foster City, CA, USA). The 6.5 mm Transwell^® ^with 8.0 μm Pore Polycarbonate Membrane Insert was purchased from Corning Incorporated (Lowell, MA, USA). BD Matrigel™ and BD Pharmingen™ Annexin V-FITC Apoptosis Detection Kit I was obtained from BD Biosciences (San Jose, CA, USA). The CellTiter-Glo^® ^Luminescent Cell Viability Assay Kit was purchased from Promega Corp. (Madison, WI, USA). The NF-κB-Met-Luc2 reporter vector was obtained from Clontech Laboratories, Inc. (Mountain View, CA, USA). 3-(4,5-Dimethylthiazol-2yl)-2,5-diphenyltetrazolium bromide (MTT) was purchased from Research Products International Corp. (Mt. Prospect, IL, USA). Cisplatin was obtained from Enzo Life Sciences, Inc. (Plymouth Meeting, PA, USA). Doxorubicin hydrochloride was purchased from Thermo Fisher Scientific (Pittsburgh, PA, USA).

### Cell lines and culture conditions

All cell lines (human breast cancer cell lines, SK-BR-3 and MCF-7) were purchased from the American Type Culture Collection, and were maintained in RPMI-1640 medium supplemented with 10% FBS, penicillin (100 unit/ml), and streptomycin (100 μg/ml). Both cell lines were cultured at 37°C in a humidified atmosphere containing 5% carbon dioxide. The culture medium was changed every other day and the cells were passaged when they reached 80 to 90% confluency.

### siRNA design and synthesis

siRNAs targeting IKKε [GenBank: NM_014002] were designed using BLOCK-iT™ RNAi Designer (Invitrogen), siRNA Target Finder (Ambion, Austin, TX, USA), siRNA Target Finder (GeneScript, Piscataway, NJ, USA) and siRNA target Designer (Promega). Eight siRNAs targeting different regions of IKKε mRNA were designed (Table 1) and were purchased from Ambion and Invitrogen. These synthetic siRNAs are of 19 nucleotides with two thymidine deoxynucleotide (T) 3' overhangs. All designed siRNA sequences were blasted against the human genome database to eliminate cross-silence phenomenon with nontarget genes. Scrambled siRNA (Ambion) that does not target any gene was used as the negative control siRNA.

**Table 1:** Sense strand sequence of IKKε siRNA [GenBank: NM_014002]

Number	Starting site	Sequence
siR-1	482	5'-GGUCUUCAACACUACCAGCtt-3'
siR-2	2538	5'-GGCAUCCUGAAGCAUUAGAtt-3'
siR-3	551	5'-GCUGAACCACCAGAACAUCtt-3'
siR-4	533	5'-GUUUGAGGUCCUGCGGAAGtt-3'
siR-5	820	5'-GCAUCUACAAGCUGACAGAtt-3'
siR-6	1960	5'-GGGAUCAGGUACAUGAGGAtt-3'
siR-7	1968	5'-GUACAUGAGGACAGAAGCAtt-3'
siR-8	1978	5'-ACAGAAGCAUCCAGCAGAUtt-3'

### Transfection of siRNA

Cells were transfected with siRNA and Lipofectamine-2000 according to the manufacturer's instructions. Briefly, cells were seeded in a 24-well-plate at a density of 0.5 × 10^5 ^cells/well with antibiotics-free medium 12 hours before the transfection. One and a half microliters of the siRNA (20 μM) were mixed with 1 μl Lipofectamine-2000 in 50 μl serum-free RPMI-1640 medium and were incubated at room temperature for 25 minutes to form a complex. After washing cells with PBS, the 50 μl transfection mixtures were added to each well with 450 μl RPMI-1640 medium containing 10% FBS at a final concentration of 50 nM siRNA. Twenty-four hours after the transfection, the medium was replaced with fresh 500 μl RPMI-1640 medium containing 10% FBS. Forty-eight hours after the transfection, cells were collected for RNA and protein isolation.

### Real-time RT-PCR

Total RNA was isolated from cells using TRIzol reagent according to the manufacturer's protocol. Total RNA (200 ng) was converted to cDNA using random hexamer primer and MultiScribe Reverse Transcriptase Reagent. One hundred nanograms of cDNA were amplified by real-time PCR using SYBR Green-1 dye universal Master mix on an ABI Prism 5700 Sequence Detection System (Applied Biosystems). To confirm the PCR specificity, PCR products were subjected to a melting-curve analysis. The comparative threshold method was used to calculate the relative amount of mRNA of treated sample in comparison with control samples [14,15]. The primers used for the study included: IKKε, 5'-ACTCTGGAAGTGGCAA GGACAT-3' (forward) and 5'-TACCTGATCCCGGCTCTTCACCA-3' (reverse); IKKα, 5'-TCT GGAACAGCGTGCCATTGATCT-3' (forward) and 5'-ATTACTGAGGGCCACTTCCACCTT-3' (reverse); IKKβ, 5'-ACTGGAGCAGCAGAAGTACACAGT-3' (forward) and 5'-ATCAG CATCAGTTGCAGCCACTTC-3' (reverse); TBK1, 5'-AGGATTGCCTGATCCAGCCAAGAT-3' (forward) and 5'-CCACTGGACGAAGGAAGCTTATGC-3' (reverse); and Bcl-2, 5'-AGGCAT GTTGACTTCACTTGTGGC-3' (forward) and 5'-GCATGCGGCCTCTGTTTGATTTCT-3' (reverse). We used 18s ribosomal RNA as an internal control, and the primers were 5'-GTCTGTGATGCCCTTAGATG-3' (forward primer), and 5'-AGCTTATGACCCGCACTT AC-3' (reverse primer).

### Western blotting

The cultured cells were washed twice with ice-cold PBS and lysed on ice in RIPA lysis buffer containing freshly added protease and phosphatase inhibitor cocktails. After 5 minutes of incubation, the cell lysate was collected by centrifugation at 4°C for 10 minutes at 12,000 rpm. The amount of total protein was determined using a BCA protein assay kit (Pierce, Rockford, IL, USA). An equal amount of total protein (20 μg) was loaded and separated by SDS-PAGE. The protein was transferred to a nitrocellulose membrane, blocked and probed with appropriate antibodies. The protein was then visualized using horseradish peroxidase-conjugated secondary antibodies and the FluorChem FC2 imaging system (Alpha Innotech, San Leandro, CA, USA). Anti-IKKε/IKKi antibody (Sigma-Aldrich), anti-β-Actin antibody (Rockland, Gilbertsville, PA, USA), anti-Bcl2 antibody (Abcam, Cambridge, MA, USA), anti-cyclin D_1 _(Abcam) and horseradish peroxidase-conjugated secondary antibody (Invitrogen) were used in the western blotting assay.

### Focus formation assay

Forty-eight hours after the transfection, 5 × 10^3 ^MCF-7 cells/well or 7.5 × 10^3 ^SK-BR-3 cells/well were seeded in six-well plates. The medium was changed every 2 days. Cells cultured for 9 days were washed twice with ice-cold medium, fixed by ice-cold methanol, and stained with 0.2% crystal violet. Images of the colonies were obtained using a digital camera.

### Soft agar assay

Colony formation ability was examined by anchorage-independent soft agar assay on MCF-7 cells. Briefly, 1.5 ml FBS supplemented medium containing 0.5% agarose were added in 35-mm cell culture dishes and allowed to solidify (base agar). Next, 1 × 10^4 ^siRNAs transfected MCF-7 cells were mixed with 1.5 ml FBS-supplemented medium containing 0.35% agarose and added to the top of base agar. The cells were then cultured for 14 days at 37°C under 5% carbon dioxide. The dishes were stained with 0.005% crystal violet, and the colonies were examined with microscope and digital camera.

### Wound healing assay

SK-BR-3 cells seeded in 12-well plates (2 × 10^5 ^cells/well) were transfected with 50 nM siRNA as described above. Once the cells reached 90% confluency, a wound area was carefully created by scraping the cell monolayer with a sterile 10 μl pipette tip. The cells were then washed once with Dulbecco's PBS to remove detached cells. Subsequently, the cells were incubated at 37°C in 5% carbon dioxide. The width of the wound area was monitored with an inverted microscope at various time points. The normalized wound area (wound area_48 hours_/wound area_0 hours_) was calculated using the software TScratch [16].

### Migration assay and invasion assay

We evaluated the effect of IKKε siRNA on invasiveness properties of breast cancer cells using transwell migration and invasion assays. Forty-eight hours after the transfection, SK-BR-3 cells or MCF-7 cells were trypsinized and resuspended in FBS-free RPMI-1640 medium. For the migration assay, a total of 1 × 10^5 ^cells were plated in the top chamber of the transwell with a noncoated polycarbonate membrane (6.5 mm diameter insert, 8.0 μm pore size; Corning Incorporated). For the invasion assay, 1 × 10^5 ^cells were plated in the top chamber of the transwell with a matrigel-coated polycarbonate membrane. RPMI-1640 medium with 10% FBS was added to the lower chamber as a chemoattractant. After incubation for 48 hours (migration assay) or 60 hours (invasion assay), cells on the lower surface of the membrane were fixed with 10% formalin and stained with 0.2% crystal violet. Cells that did not migrate through the pores were mechanically removed by a cotton swab [17]. The images of migrated cells were acquired by an inverted microscope with a magnification of 200×. The number of migrated or invaded cells was counted from five or six randomly selected fields in a blind way.

### Cell proliferation assay

The effect of siRNA on cell proliferation was measured using the CellTiter-Glo^® ^Luminescent Cell Viability Assay Kit (Promega) according to the manufacturer's protocol. Briefly, SK-BR-3 cells (5,000 cells/well) or MCF-7 cells (2,500 cells/well) seeded in 96-well plates were transfected with 50 nM siRNA as described above. Seventy-two hours and 120 hours after the transfection, 100 μl CellTiter-Glo^® ^reagent was added to each well that contained 100 μl cell culture medium. Cells were then lysed by shaking in an orbital shaker for 2 minutes, followed by incubation at room temperature for 10 minutes to stabilize the luminescent signal. The luminescent intensity was measured using a Beckman DTX 880 multimode Detector (Beckman coulter, Inc., Brea, CA, USA).

### NF-κB transcriptional activity assay

The transcriptional activity of NF-κB was examined using a Ready-To-Glow™ secreted luciferase reporter system, NF-κB-Met-Luc2, which contains the NF-κB promoter element upstream of the luciferase gene. The expression of luciferase was used to monitor the activity of NF-κB. Fifty thousand SK-BR-3 cells or MCF-7 cells were seeded in 24-well plates and transfected with siRNAs. Twenty-four hours after siRNA transfection, the cells were co-transfected with NF-κB-Met-Luc2 reporter vector and β-galactosidase reporter vector (used as an internal control). The culture medium was collected 24 hours post-transfection to measure the luciferase activity. The cells were lysed with reporter lysis buffer and the β-galactosidase activities of whole cell lysate were measured. The relative luciferase activity was calculated by normalizing results with the β-galactosidase expression.

### Cell cycle assay and apoptosis assay

Forty-eight hours after the siRNA transfection, the cells were collected and fixed with ice-cold 70% ethanol. Before staining, the cells were washed with Dulbecco's PBS and incubated with propidium iodide/RNase staining buffer for 30 minutes at room temperature. Cell cycle analysis was carried out with a FACSCalibur Flow cytometer (BD Biosciences). To analyze apoptosis, cells were collected 72 hours post-transfection, and then stained with Annexin V-FITC and propidium iodide using the Annexin V-FITC Apoptosis Detection Kit I. The percentage of apoptotic cells was quantified by a FACSCalibur Flow cytometer. Paclitaxel (100 nM, 24-hour incubation) was used in the apoptosis assay as an apoptosis inducer to validate the measurements.

### Combinational treatment of cells with siRNA and chemotherapy agents

Cells (10,000 cells/well) were seeded in a 96-well plate and transfected with siRNA as described above. Twenty-four hours after the transfection, the cells were incubated with medium supplemented with serial concentrations of cisplatin and doxorubicin for another 24 hours. Untransfected cells treated with different concentration of cisplatin or doxorubicin are defined as medium control group. Cell viability was then determined by MTT assay. MTT in PBS was added into cells at a final concentration of 0.5 mg/ml. After 1 hour of incubation at 37°C, the medium was aspirated and 100 μl dimethylsulfoxide was added to dissolve the cells and the absorbance was measured at 570 nm.

### Statistical analysis

Data were expressed as the mean ± standard deviation. Difference between any two groups was determined by analysis of variance. *P *< 0.05 was considered statistically significant.

## Results

### Silencing of IKKε gene by predesigned siRNAs

**Figure 1:**
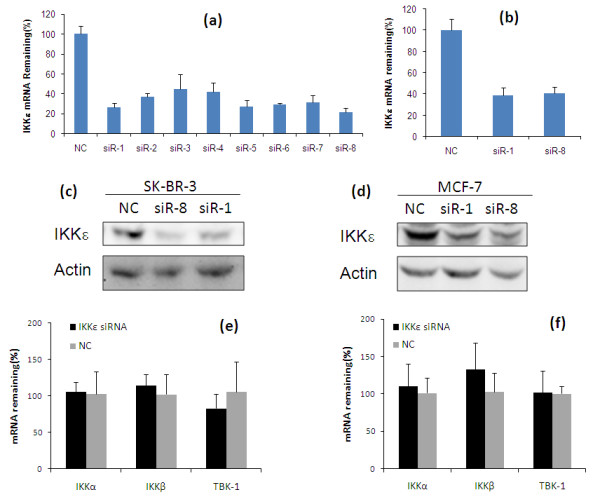
**Silencing effect of predesigned IKKε siRNAs in SK-BR-3 and MCF-7 cells**. **(a) **SK-BR-3 cells were transfected with eight predesigned IKKε siRNAs (siR-1 to siR-8) and negative control siRNA (NC) at a concentration of 50 nM. Cells were harvested 48 hours after the transfection, and the silencing effect at the IKKε mRNA level was determined using real-time RT-PCR. **(b) **MCF-7 cells were transfected with selected siR-1, siR-8, and NC. The Silencing effect at the IKKε mRNA level was measured using real-time RT-PCR. The silencing effect of IKKε siRNA at the protein level was determined using western blot in **(c) **SK-BR-3 cells and **(d) **MCF-7 cells. The effect of IKKε suppression on IKKα, IKKβ and TBK1was examined in **(e) **SK-BR-3 cells and **(f) **MCF-7 cells.

To silence IKKε expression, we designed up to eight siRNAs (Table 1) targeting different mRNA regions of IKKε. Silencing effects of these predesigned IKKε siRNAs were examined in SK-BR-3 cells at a concentration of 50 nM after complexation with Lipofectamine-2000. A scrambled siRNA that does not target any gene was used as the negative control. All eight siRNAs showed a significant silencing effect (*P *< 0.05) and knocked down 55.2 to 77.9% of IKKε mRNA in comparison with scrambled siRNA (Figure 1a). Among them, siR-1 and siR-8 showed the greatest suppression of IKKε and therefore these two siRNAs were selected for subsequent biological studies. Considering the fact that siRNA transfection efficiency may vary in different cell lines, we also examined the silencing effects of siR-1 and siR-8 in MCF-7 cells. Approximately 61.3% and 59.0% of IKKε mRNA were silenced in MCF-7 cells after treatment with siR-1 and siR-8 (Figure 1b), respectively. The silencing effect of IKKε expression at the protein level was also confirmed with western blot. As shown in Figure 1c, both siR-1 and siR-8 significantly inhibited the IKKε protein expression in SK-BR-3 cells and MCF-7 cells, which is consistent with the silencing effect at the mRNA level.

**Figure 2:**
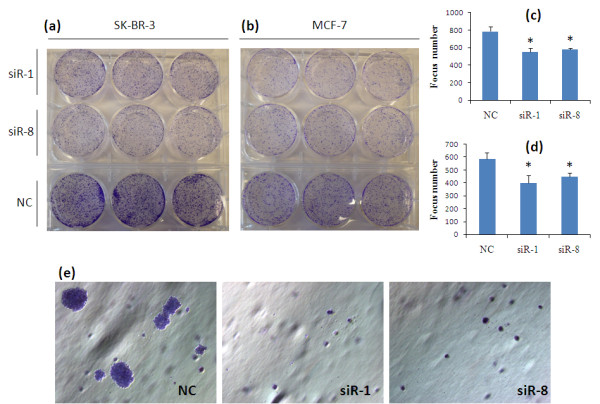
**Silencing of IKKε inhibits focus formation and colony formation**. Forty-eight hours after the siRNA transfection, **(a) **SK-BR-3 cells and **(b) **MCF-7 cells were seeded in six-well plates, and the medium was changed every 2 days. Cells cultured for 9 days were washed twice with ice-cold medium, fixed with ice-cold methanol, and stained with 0.2% crystal violet. Images of the colonies were obtained with a digital camera. **(c), (d) **The focus number was counted and the result represented as mean ± standard deviation (*n *= 3). **(e) **A soft agar assay was conducted to examine the colony formation ability of MCF-7 cells. Twenty-four hours after the siRNA transfection, MCF-7 cells were seeded in 0.35% agarose in RPMI-1640-supplemented 10% FBS at a density of 1 × 10^4 ^per 35-mm culture dish and allowed to grow for 14 days. Results are representative picture of colonies of two independent experiments done in triplicate. *P < 0.05. NC, negative control siRNA.

To study whether IKKε silencing upregulates other IKK kinases, we assayed the mRNA expression of IKKα, IKKβ and TBK1 after silencing IKKε in MCF-7 cells and SK-BR-3 cells (Figure 2e, f). In both cell lines, IKKε suppression did not significantly influence the expressions of IKKα, IKKβ and TBK1 (*P *> 0.05).

### Silencing of IKKε inhibits focus formation of breast cancer cells

First, we used a focus formation assay to test whether silencing IKKε in breast cancer cells affects the clonogenic potential, which correlates with tumor formation *in vivo *[18]. Forty-eight hours after the transfection, a single-cell suspension was seeded into six-well plates and incubated for 9 days to allow focus formation. The cells' foci were fixed, stained with crystal violet, and counted. As Figure 2a shows, SK-BR-3 cells treated with IKKε siRNA exhibited smaller focus diameter as well as focus numbers compared with cells treated with the scrambled siRNA. Similar results were also observed in MCF-7 cells (Figure 2b). These data indicated that inhibition of IKKε significantly decreases the cells' focus formation potential, which correlates with the formation of tumors in nude mice [19].

### Silencing IKKε inhibits anchorage-independent growth of breast cancer cells

Anchorage-independent growth capability is one of the important characteristics of oncogenically transformed cells. In order to examine whether IKKε knockdown can influence the anchorage-independent growth potential, we performed a soft agar assay in MCF-7 cells. Twenty-four hours after the transfection, a single-cell suspension was seeded into 0.35% agarose supplemented with RPMI-1640 medium and 10% FBS. The cells were cultured for another 14 days under normal cell culture conditions to allow colony formation. As shown in Figure 2e, silencing IKKε in MCF-7 cells dramatically inhibited the transformed phenotype. Individual colony size was much smaller in IKKε siRNA transfected cells compared with negative control siRNA-treated cells. This result indicated that silencing of IKKε in breast cancer cells suppress anchorage-independent growth capability.

### Silencing of IKKε inhibits cell motility and invasion

**Figure 3:**
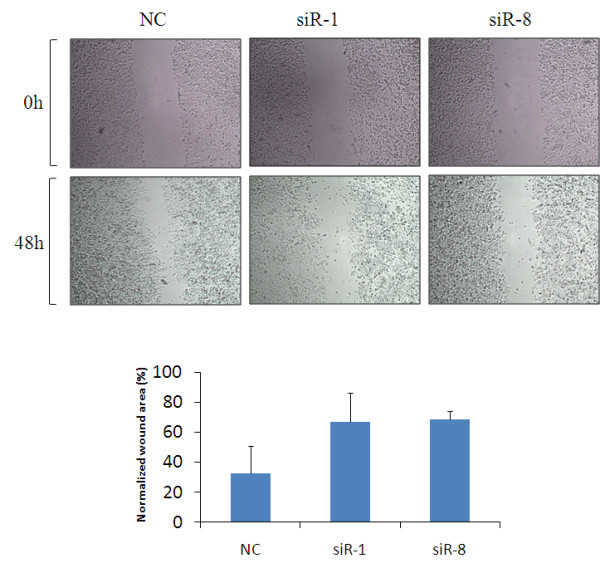
**Study of cell motility by wound healing assay**. A wound healing assay was used to evaluate the migration of SK-BR-3 cells after silencing IKKε. Fifty-six hours after the transfection of siRNA, cells were wounded and monitored with a microscope every 12 hours. The migration was determined by the rate of cells filling the scratched area. The normalized wound area was calculated by the software TScratch [16]. Similar results were obtained in three independent experiments. NC, negative control siRNA.

Decreased clonogenic potential is usually associated with the loss of invasion capabilities in tumor cells [18]. The cell motility of breast cancer cells was therefore tested using a classic wound healing assay in which the cell monolayer was scratched and cells migrating to the wound area were monitored at different time points. Compared with cells transfected with scrambled siRNA, the cells treated with siR-1 and siR-8 showed a wider wound area 48 hours after wound generation, and took a longer time to fill the wound area, indicating a defect in migration (Figure 3).

**Figure 4:**
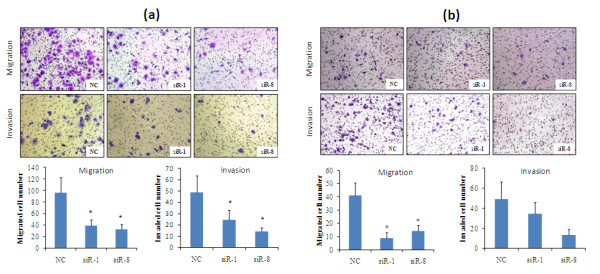
**Silencing of IKKε expression inhibits migration and invasion abilities of SK-BR-3 cells and MCF-7 cells**. Cell migration was determined using Boyden transwell chambers. Forty-eight hours after the transfection with siRNA, **(a) **SK-BR-3 cells and **(b) **MCF-7 cells were suspended in serum-free medium and seeded on 24-well transwell plates. RPMI-1640 with 10% FBS was incubated in the lower chamber as the chemoattractant. Cells migrated though pores to the bottom surface of the transwell were fixed with 10% buffered formalin, stained with 0.2% crystal violet and counted. Six random microscopic fields were counted for each group. Cell invasion was assayed in transwell coated with Matrigel. Cells crossed the Matrigel-coated filter were fixed, stained and counted. Representative pictures of the bottom surface are shown. Six random microscopic fields were counted for each group. The results presented are an average of six random microscopic fields from three independent experiments. Significant reduction of migration and invasion was observed after silencing IKKε expression in SK-BR-3 cells and MCF-7 cells. *P < 0.05. NC, negative control siRNA.

Since both cell migration and invasion are critical properties for the spreading of cancer cells and metastases, we further investigated the cell invasiveness using *in vitro *migration and invasion assays. Migration assay using uncoated Boyden chamber is a common method to examine the *in vitro *migration ability of tumor cells. Cells that migrated to the bottom of the transwell were fixed, stained and counted. Compared with the control group, IKKε siRNA transfected cells showed a significant decrease in the number of migrated cells in MCF-7 cells and SK-BR-3 cells (Figure 4a, b). Additionally, matrigel-coated transwell chambers were used to access the invasive capacities of breast cancer cells. Consistent with the finding in migration assay, cells treated with IKKε siRNA demonstrated significant reduction in cell invasion ability by 50 to 70% in SK-BR-3 cells and 30 to 73% in MCF-7 cells in comparison with scrambled siRNA-treated cells (Figure 4a, b). Taken together, these results indicate that silencing of IKKε decreases the invasive properties of breast cancer cells.

### Silencing of IKKε inhibits the proliferation of breast cancer cells

**Figure 5:**
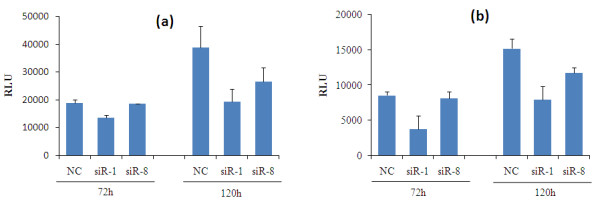
**Effect of IKKε siRNA on cell proliferation**. **(a) **SK-BR-3 cells and **(b) **MCF-7 cells were transfected with siR-1, siR-8, and negative control siRNA (NC). Cell growth was assayed at 72 hours and 120 hours post-transfection using the CellTiter-Glo^® ^Luminescent Cell Viability Assay Kit. Result represented as mean ± standard deviation (*n *= 3). RLU, Relative Luminescence Unites.

Since oncogene is known to facilitate tumor cell growth, we next examined the proliferation of breast cancer cells after silencing of IKKε with siRNA. Cell growth was determined at 72 hours and 120 hours post-transfection. Compared with cells transfected with the scrambled siRNA, cells treated with IKKε siRNAs demonstrated slower growth rate and lower viability (Figure 5). This observation is in accordance with the finding that a lentiviral shRNA targeting IKKε suppressed the proliferation and viability of MCF-7 cells [6]. These results suggested the pivotal role of IKKε in the proliferation and survival of breast cancer cells, and suppression of IKKε could lead to inhibition of cell proliferation. Moreover, the inhibition effect on cell proliferation is more significant at 120 hours rather than 72 hours post-transfection.

### Silencing of IKKε induces cell arrest in G_0_/G_1 _phase

**Figure 6:**
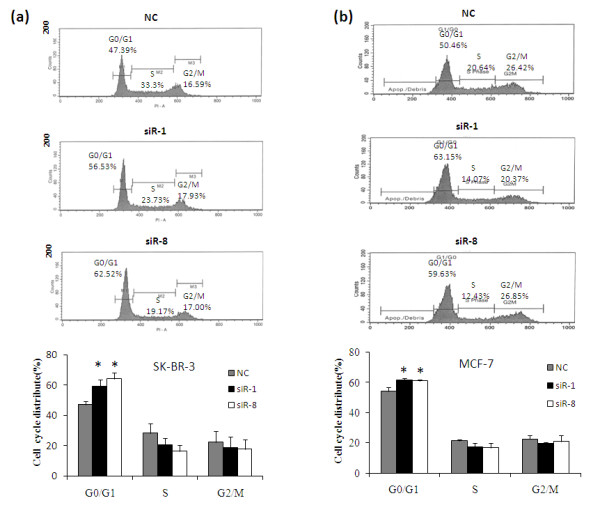
**Silencing of IKKε leads to G_0_/G_1 _phase arrest**. Cell cycle distribution of **(a) **SKBR-3 cells and **(b) **MCF-7 cells treated with 50 nM siR-1, siR8, and scrambled siRNA were accessed by flow cytometry 48 hours post-transfection. Results are representative of three independent experiments, represented as mean ± standard deviation (*n *= 3). NC, negative control siRNA.

To identify the mechanism for this anti-proliferation effect, we investigated the cell cycle distribution of breast cancer cells after the silencing of IKKε. As Figure 6 shows, cells transfected with IKKε siRNA induced a significant G_0_/G_1 _block in comparison with cells treated with scrambled siRNA. This was accompanied by a reduction of the proportion of M-phase cells, while there was little difference in the G_2_/M distribution. The G_0_/G_1 _distribution of SK-BR-3 cells transfected with IKKε siR-1 and siR-8 was 59.2% and 64.4%, respectively, in comparison with 50.4% in cells treated with scrambled siRNA (Figure 6a). In the same experiment, a similar result was observed in MCF-7 cells (Figure 6b). The percentages of cells in the G_0_/G_1 _phase were 61.1% and 61.8% for cells treated with siR-1 and siR-8, respectively. In comparison, only 54.3% of MCF-7 cells treated with scrambled siRNA were in the G_0_/G_1 _phase. The data revealed that IKKε siRNA inhibits cell proliferation via blocking cell cycle progression at the G_0_/G_1 _phase.

### Silencing of IKKε induces negligible apoptosis

**Figure 7:**
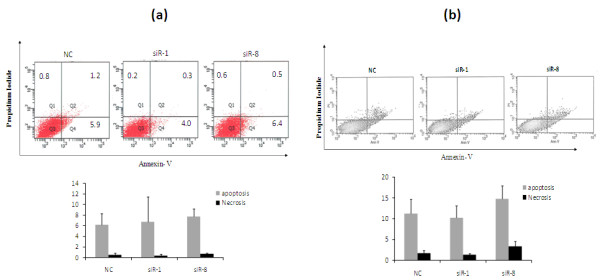
**Effect of IKKε siRNA on apoptosis of breast cancers**. **(a) **SK-BR-3 cells or **(b) **MCF-7 cells were transfected with siR-1, siR-8, and scrambled siRNA at 50 nM. Seventy-two hours after transfection, cells were stained with Annexin-V-FITC and propidium iodide followed by flow cytometry. The percentage of early apoptotic (right bottom), late apoptosis (right top), and necrotic cells are shown. Three independent experiments were conducted. NC, negative control siRNA.

Flow cytometry was next used to assay the apoptosis of breast cancer cells after inhibition of IKKε using siRNA. No significant difference of Annexin-V-positive apoptotic cells was observed in the IKKε siRNA-treated group in comparison with cells transfected with scrambled siRNA. As Figure 7 indicated, IKKε-specific siRNA, siR-1 and siR-8, induced apoptosis in 4.0% and 6.4% of SK-BR-3 cells, respectively, while the scrambled siRNA induced apoptosis in 5.9% of cells (*P *= 0.821). In MCF-7 cells, siR-1, siR-8, and scrambled siRNA induced apoptosis in 10.2%, 14.7% and 11.2% of cells respectively (*P *= 0.266). No significant difference was observed in this study, suggesting that knockdown of IKKε alone may not induce apoptosis of breast cancer cells.

### Silencing of IKKε decreases the basal activity of NF-κB

**Figure 8:**
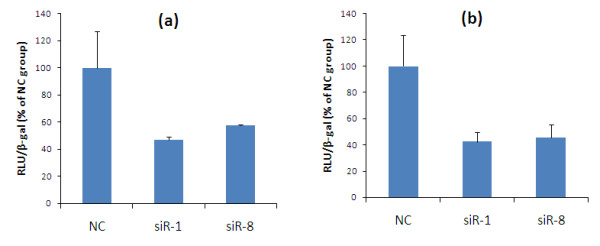
**Silencing of IKKε expression reduces basal NF-κB transcriptional activity in SK-BR-3 cells and MCF-7 cells**. Twenty-four hours after the siRNA transfection, **(a) **SK-BR-3 cells and **(b) **MCF-7 cells were co-transfected with the NF-κB Met Luc2 reporter vector, which contains the NF-κB promoter element upstream of the luciferase gene, and the β-galactosidase reporter vector as an internal control. The expression of luciferase was used to monitor the transcriptional activity of NF-κB. The relative luciferase activity was normalized with the β-galactosidase expression. Result representative of three independent experiments. * *P *< 0.05. NC, negative control siRNA; RLU, Relative Luminescence Unites.

To determine whether the knockdown of IKKε gene affects the constitutive NF-κB activity in breast cancer cells, the NF-κB-dependent luciferase reporter assay was performed. Cells were transfected with siRNA for 24 hours, followed by co-transfection with the NF-κB-MetLuc2 reporter vector and the β-galactosidase reporter vector, which was used as an internal control to normalize the transfection efficiency. As shown in Figure 8, the NF-κB transcriptional activity was reduced in cells treated with IKKε siRNA in comparison with cells treated with scrambled siRNA. In MCF-7 cells, the NF-κB basal level in IKKε siRNA-treated cells decreased to around 42 to 46% of the control group. Similar result was also observed in SK-BR-3 cells, where the NF-κB basal level decreased to approximately 47 to 58% of the control group upon IKKε silencing. This result suggests that IKKε may play an important role in controlling the NF-κB dependent activity in breast cancer cells. This is in agreement with the finding that IKKε activates the NF-κB pathway in breast cancer, although the mechanism is not fully elucidated [6,11].

### Silencing of IKKε regulates NF-κB-related downstream genes

**Figure 9:**
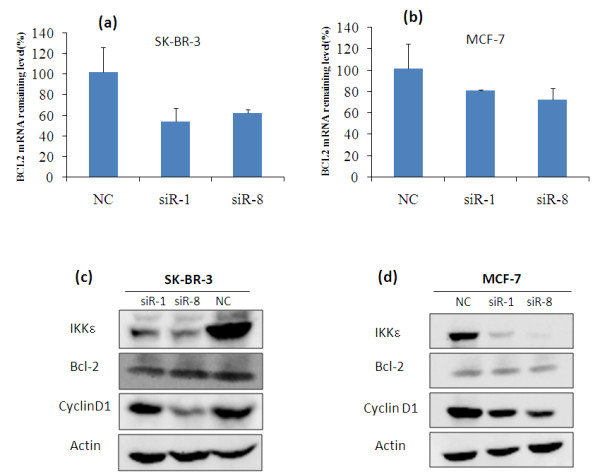
**Silencing of IKKε leads to reduction of Bcl-2 and cyclin D_1 _in breast cancer cells**. The Bcl-2 mRNA level was determined in **(a) **SK-BR-3 cells and **(b) **MCF-7 cells using real-time RT-PCR. The relative levels of Bcl-2 mRNA in the scrambled siRNA group were normalized as 100%. Results represented as mean ± standard deviation (*n *= 3). IKKε, Bcl-2 and cyclin D_1 _expressions at the protein level were assayed using western blot in IKKε siRNA-transfected **(c) **SK-BR-3 cells and **(d) **MCF-7 cells. NC, negative control siRNA.

It is reported that breast cancer cells overexpressing IKKε showed increased expression of Bcl-2 compared with cells without IKKε overexpression [6]. The Bcl-2 expression levels in SK-BR-3 cells and MCF-7 cells were therefore examined after the inhibition of IKKε. As indicated in Figure 9a, b, the Bcl-2 mRNA level decreased in both SK-BR-3 cells and MCF-7 cells after the treatment with siR-1 and siR-8. This is in accordance with a previous finding that suppression of the IKKε gene resulted in downregulation of Bcl-2 expression [6]. We also examined the protein level of Bcl-2 using western blot (Figure 9c, d). Consistent with the mRNA results, both SK-BR-3 cells and MCF-7 cells showed reduction of Bcl-2 protein expression after the IKKε siRNA treatment. Image J software was used to normalize the Bcl-2 expression with β-actin. In SK-BR-3 cells, the normalized Bcl-2 expressions of siR-1 and siR-8 siRNA treated cells were 40% and 66%, respectively, in comparison with the control group. In MCF-7 cells, the normalized Bcl-2 expressions of siR-1 and siR-8 siRNA-treated cells were 88% and 48%, respectively, in comparison with the control group.

Since cyclin D_1 _was reported as the key regulatory protein for progression through the G_1 _phase of breast cancer cells [20], we next examined whether the expression of cyclin D_1 _was responsible for the G_0_/G_1 _cell cycle arrest in IKKε siRNA-treated cells. As Figure 9c, d indicates, silencing of IKKε significantly decreased the expression of cyclin D_1 _in breast cancer cells. In SK-BR-3 cells, the normalized Bcl-2 expressions of siR-1 and siR-8 siRNA-treated cells were 64% and 34%, respectively, in comparison with the control group. In MCF-7 cells, the normalized Bcl-2 expressions of siR-1 and siR-8 siRNA-treated cells were 71% and 45%, respectively, in comparison with the control group. Overexpression of cyclin D_1 _has been shown to shorten the G_1 _phase, and subsequently increase the cell proliferation [21]. This result therefore suggests that cyclin D_1 _is an important mediator in the oncogenic role of IKKε in breast cancer.

**Figure 10:**
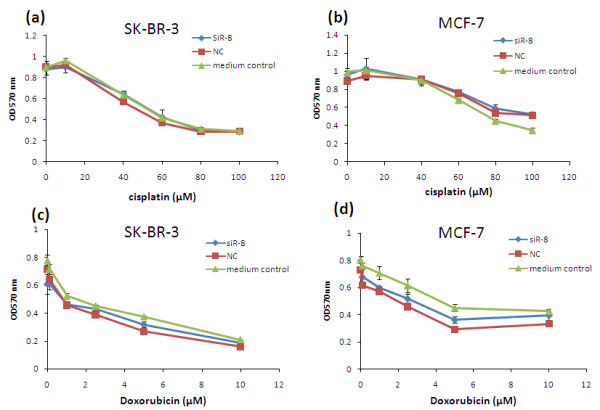
**Knockdown of IKKε does not sensitize SK-BR-3 cells and MCF-7 cells to cisplatin and doxorubicin**. Twenty-four hours after the siRNA transfection, **(a), (c) **SK-BR-3 cells and **(b), (d) **MCF-7 cells were treated with indicated amounts of (a), (b) cisplatin or (c), (d) doxorubicin for 24 hours, and the cell viability was measured by 3-(4,5-dimethylthiazol-2yl)-2,5-diphenyltetrazolium bromide (MTT) assay. Results represented as mean ± standard deviation (*n *= 3). NC, negative control siRNA.

Inhibition of NF-κB has been reported to sensitize breast cancer cells to doxorubicin [22]. Overexpression of IKKε is associated with cell resistance to cisplatin in ovarian cancer. Silencing of IKKε sensitized ovarian cancer cells to cisplatin-induced apoptosis and cell death [23]. Moreover, IKKε is an important mediator that protects cells from DNA-damage-induced cell death [24]. Therefore it would be interesting to evaluate whether IKKε silencing can sensitize the response of breast cancer cells to chemotherapy reagents. Herein we investigated the response of breast cancer cells to cisplatin and doxorubicin after silencing IKKε. MCF-7 cells and SK-BR-3 cells were transfected with 50 nM IKKε siRNA or scrambled siRNA, followed by incubation with different concentrations of cisplatin or doxorubicin. After 24 hours of incubation, cell viability was measured by the MTT assay. As Figure 10 showed, IKKε suppression did not sensitize breast cancer cells to cisplatin (10 to 100 μM) (Figure 10a, b) and doxorubicin (0.1 to 10 μM) (Figure 10c, d).

## Discussion

The NF-κB pathway plays an important role in immune response, inflammation, and cancer development [25]. As a recently indentified kinase in the NF-κB pathway, IKKε is upregulated in a great proportion of breast cancer cells as well as tumor specimens [6]. Our findings support the hypothesis that IKKε plays an important role in the tumorigenesis of breast cancer.

IKKε plays an important role in cell transformation, and activation of the NF-κB pathway is involved in the IKKε-mediated transformation [6]. The tumor suppressor CYLD is directly phosphorylated by IKKε at serine-418 to decrease its deubiquitinase activity, which is essential to the IKKε-induced transformation [7]. Moreover, breast cancer cells Hs578T stably expressing IKKε K38A (kinase-inactive IKKε) showed dramatically low colony formation ability in soft agar compared with cells transfected with the control vector (pCDNA3-FLAG-IKKε) [11]. Consistent with these observations, we found that silencing of IKKε with siRNA led to significant reduction in focus formation in both MCF-7 cells and SK-BR-3 cells (Figure 2).

Several lines of evidence implicate that NF-κB and NF-κB-related IKKs are involved in cell invasion and tumor metastasis [26,27]. For example, prevention of IKKα activation resulted in inhibition of prostate cancer metastasis in TRAMP mice [28]. For the first time, we conducted numerous experiments including the would-healing assay, migration assay, and invasion assay to assess the effect of IKKε siRNA on invasiveness properties of breast cancer cells. As shown in Figures 3 and 4, the invasiveness properties were significantly inhibited in cells treated with the IKKε siRNA in comparison with cells treated with the scrambled siRNA. These data are consistent with a previous report that breast cancer cells (NF639) transfected with IKKε K38A (kinase-inactive) vectors induced a less invasive phenotype compared with cells transfected with vectors expressing the active IKKε [11].

Recent studies have shown that IKKε knockdown with lentiviral shRNA inhibited the proliferation and survival of transformed breast cancer cells, but not the nontransformed human mammary epithelial cells (MCF-10A) [6]. A similar inhibition effect on cell proliferation was also observed in IKKε knockdown Hela cells and ovarian cancer cells [4,23]. In agreement with these findings, we observed a significant anti-proliferation effect of IKKε siRNA in breast cancer cells (Figure 5). To further elucidate the mechanism of this anti-proliferation effect, cell cycle analysis was conducted. A significant cell cycle arrest in the G_0_/G_1 _phase was observed (Figure 6). All these data strongly suggest the role of IKKε in breast cancer proliferation.

We next examined the effect of IKKε on cell apoptosis. There is some controversy regarding the role of IKKε in cell apoptosis. It has been reported that IKKε inhibition induces apoptosis in Hela cells [29]. Another report using lentiviral shRNA targeting IKKε, however, did not show any apoptosis in ovarian cancer cells (A2780). Instead, overexpression of IKKε was found associated with cisplatin resistance. Significant apoptosis was detected in IKKε knockdown A2780 cells after 20 hours of exposure to cisplatin in comparison with cells treated with cisplatin alone [23]. In the current study, we did not observe significant apoptosis in IKKε knockdown SK-BR-3 and MCF-7 cells after silencing IKKε using siRNA.

Although the relationship between IKKε and NF-κB is not fully understood, it was postulated that a significant fraction of NF-κB activation was induced by aberrant IKKε expression in tumor cells [4,6,7]. Using the NF-κB transcriptional activity assay, we showed a significant reduction in basal NF-κB activity after IKKε suppression (Figure 8). This result is in agreement with a previous finding that IKKε knockdown in Hela cells reduced constitutive activity of the NF-κB dependent promoter 3X-κB [4]. The correlation of IKKε with NF-κB may explain the role of IKKε in malignant transformation and invasiveness of tumor cells.

Moreover, we examined the expression of Bcl-2 and cyclin D_1_, which are two important proteins regulated by the NF-κB pathway. Bcl-2 is an important apoptosis regulator involved in processing multiple death signals that are associated with mitochondria [30]. The Bcl-2 expression level correlates with chemotherapy resistance [31-33]. Downregulation of Bcl-2 results in induction of apoptosis and increased sensitivity to chemotherapy drugs [34,35]. Knockdown of Bcl-2 in MCF-7 cells using siRNA, however, only increased apoptosis by 9% (at 72 hours) and 11% (at 96 hours) in comparison with the control group [36]. In addition, Akar and colleagues demonstrated that cell death (MCF-7 cells) triggered by Bcl-2 siRNA was caused by the induction of autophagic cell death rather than apoptosis. The authors did not observe any apoptosis effect in breast cancer cells upon Bcl-2 silencing [37]. These controversial reports suggested that downregulation of the anti-apoptosis protein Bcl-2 alone does not necessarily result in apoptosis, especially considering the fact that induction of apoptosis is determined by a balance of multiple pro-apoptosis proteins and anti-apoptosis proteins [38]. Similar to these findings, we only observed negligible apoptosis in breast cancer cells (Figure 7), although the Bcl-2 level was downregulated by the IKKε siRNA (Figure 9a to 9c). These results might be explained by a compensation of other existed anti-apoptosis factors. In addition, the treatment of IKKε siRNA did not sensitize breast cancer cells to cisplatin and doxorubicin (Figure 10), indicating that silencing IKKε alone may not be sufficient to induce cell apoptosis.

On the other hand, significant inhibition of cyclin D_1 _was observed in cells treatment with IKKε siRNA (Figure 9d). Cyclin D_1_, regulated by the NF-κB pathway, is overexpressed in more than 50% of breast cancers, and is identified as one of the most commonly upregulated proteins in breast cancer [39,40]. There is mounting evidence that cyclin D_1 _plays a critical role in breast cancer cell cycle control. The induction of cyclin D_1 _in breast cancer cells shortens the G_1 _phase and increases the number of cells that progress through the G_1 _phase, resulting in an increased proliferation [21]. It was reported that overexpression of an inactive mutant of IKKε (K38A) in Hs578T cells resulted in reduction of cyclin D_1 _[11]. A recent study showed that IKKε phosphorylates estrogen receptor α at serine-167 and subsequently transcriptionally upregulates cyclin D_1 _[41]. Our results showed that cyclin D_1 _expressions were downregulated upon IKKε knockdown in both estrogen receptor-positive (MCF-7) and estrogen receptor-negative (SK-BR-3) breast cancer cells (Figure 9c, d), and the reduced cyclin D_1 _expressions in both breast cancer cell lines were correlated with a cell cycle arrest in G_0_/G_1 _(Figure 6a, b).

## Conclusions

In summary, studies from our laboratory have shown that silencing of IKKε with siRNA resulted in significant inhibition of focus formation potential, anchorage-independent growth capability, migration, invasiveness, and proliferation in breast cancer cells. The NF-κB transcriptional activity and its downstream gene, cyclin D_1_, were inhibited by IKKε siRNAs. The anti-proliferation effect of IKKε siRNA is mediated by arresting cells in the G_0_/G_1 _phase. The present study provided the first evidence that silencing IKKε using synthetic siRNA inhibited the invasiveness and proliferation of breast cancer cells. Taken together, our findings not only indicate that IKKε can be a novel therapeutic target for breast cancer treatment, but also suggest a therapeutic potential of targeting IKKε with siRNA.

## Abbreviations

BSA: bovine serum albumin; FBS: fetal bovine serum; IκB: inhibitor of κB; IKK: IκB kinase; IL: interleukin; MTT: 3-(4,5-dimethylthiazol-2yl)-2,5-diphenyltetrazolium bromide; NF: nuclear factor; PBS: phosphate-buffered saline; PCR: polymerase chain reaction; RNAi: RNA interference; RT: reverse transcriptase; shRNA: short hairpin RNA; siRNA: small interfering RNA.

## Competing interests

The authors declare that they have a financial competing interest. The authors have submitted a patent disclosure relating to the content of this manuscript.

## Authors' contributions

KC and BQ designed the research. BQ performed the research. KC and BQ analyzed the data. KC and BQ wrote the paper. All authors read and approved the final manuscript.
